# 'Well, It's the Risk of the Unknown… Right?': A Qualitative Study of Perceived Risks and Benefits of HIV Cure Research in the United States

**DOI:** 10.1371/journal.pone.0170112

**Published:** 2017-01-25

**Authors:** Karine Dubé, Jeff Taylor, Laurie Sylla, David Evans, Lynda Dee, Alasdair Burton, Loreen Willenberg, Stuart Rennie, Asheley Skinner, Joseph D. Tucker, Bryan J. Weiner, Sandra B. Greene

**Affiliations:** 1 University of North Carolina Gillings School of Global Public Health, Chapel Hill, NC, United States of America; 2 Collaboratory of AIDS Researchers for Eradication (CARE) Community Advisory Board (CAB), Palm Springs, CA, United States of America; 3 defeatHIV CAB, Seattle, WA, United States of America; 4 Delaney AIDS Research Enterprise (DARE) CAB, Los Angeles, CA, United States of America; 5 Project Inform, Los Angeles, CA, United States of America; 6 AIDS Action Baltimore, Baltimore, MD, United States of America; 7 Multi-Decadal, Multi-CAB Member, Glendale, CA, United States of America; 8 Zephyr Long-Term Non-Progressors (LTNP) Foundation, Inc., Sacramento, CA, United States of America; 9 Department of Social Medicine, UNC Bioethics Center, Chapel Hill, NC, United States of America; 10 Duke Clinical Research Institute (DCRI), Durham, NC, United States of America; 11 UNC Institute of Global Health and Infectious Diseases (IGHID), Chapel Hill, NC, United States of America; 12 UNC Project China, Guangzhou, China; 13 Department of Global Health, University of Washington, Seattle, WA, United States of America; University of Toronto, CANADA

## Abstract

**Introduction:**

Biomedical research towards an HIV cure is advancing in the United States and elsewhere, yet little is known about perceptions of risks and benefits among potential study participants and other stakeholders. We conducted a qualitative study to explore perceived risks and benefits of investigational HIV cure research among people living with HIV (PLWHIV), biomedical HIV cure researchers, policy-makers and bioethicists.

**Methods:**

We conducted a qualitative research study using in-depth interviews with a purposive sample of PLWHIV, biomedical HIV cure researchers, policy-makers and bioethicists in 2015–2016. We analysed interview transcripts using thematic analysis anchored in grounded theory.

**Results:**

We conducted and analyzed 36 key informant interviews. Qualitative analysis revealed four main findings. 1) Potential HIV cure study volunteers noted needing more information and education about the potential risks of HIV cure research. 2) Biomedical HIV cure researchers, policy-makers and bioethicists showed less awareness of social and financial risks of HIV cure research than PLWHIV. 3) Most respondents across the different categories of informants identified some risks that were too great to be acceptable in HIV cure research, although a subset of PLWHIV did not place an upper limit on acceptable risk. 4) PLWHIV showed a better awareness of potential psychological benefits of participating in HIV cure research than other groups of stakeholders.

**Conclusion:**

Our research suggests that PLWHIV have a variable understanding of the individual risks, sometimes substantial, associated with participating in biomedical HIV cure research studies. Community engagement and increased research literacy may help improve community understanding. Intensive informed consent procedures will be necessary for ethical study implementation. The current state of HIV cure research offers greater potential benefits to society than to participants. There is likely to be disagreement among regulators, researchers, clinicians, and potential participants about what constitutes acceptable risk for HIV cure studies.

## Introduction

Most HIV cure studies ask participants to undertake substantial individual risk with little to no corresponding individual and direct clinical benefit [1]. Especially in the context of highly effective modern combined antiretroviral therapy (cART), biomedical HIV cure research that includes treatment interruption may confer risk of viral rebound that could be avoided by the current standard of care [2]. As revealed from a previous quantitative study in people living with HIV (PLWHIV) about cure research [3], there may be psychological and emotional attachments to the benefits of current HIV treatment. PLWHIV place value on how HIV treatment reduces the risk of passing on the virus to one’s sex partner(s) and fear losing viral suppression achieved with newer antiretroviral drugs, particularly if viral suppression was previously impossible with older drugs [3].

The Food and Drug Administration (FDA) defines HIV cure research “as any investigation that evaluates: [1] a therapeutic intervention (or approach) that controls or eliminates HIV infection to the point that no further medical interventions are needed to maintain health; and [2] preliminary scientific concepts that might ultimately lead to such a therapeutic intervention” [4]. Two main approaches are being investigated: 1) a sterilizing cure, which would clear all latent viral reservoirs in the body (eradication); and 2) a functional cure, which would durably suppress HIV without ongoing treatment through a variety of potential mechanisms [5]. A functional cure, or a long-term viral suppression, may be easier to achieve than a complete sterilizing cure. Early phase studies suggest the need for multiple approaches to achieve durable suppression [6]. There are many different HIV cure modalities under investigation, some re-purposing drugs borrowed from oncology and others developing new substances and approaches that have substantial or unknown risks. In 2016, there were more than 125 ongoing or completed HIV cure-related clinical studies around the world, primarily in the United States [7]. A better understanding of the perceptions and attitudes toward risks and benefits of HIV cure research by those designing, approving, and participating in studies could help ensure the ethical design and conduct of research studies and increase the odds of study success.

To address the lack of empirical data in the literature regarding acceptability of HIV cure research, we implemented a qualitative study to explore the perceptions of risks and benefits among three types of stakeholders in the United States: (1) PLWHIV, (2) biomedical HIV cure researchers, and (3) policy-makers or bioethicists. Given the complexity of HIV cure research and the diversity of stakeholders involved in implementing HIV cure science[8], it was important to solicit input from different groups of informants. We inquired about perceptions of benefits and risks, including unacceptable risks. Data inform possible perceived risk thresholds and identify features that could make participants reluctant to join HIV cure studies, researchers reluctant to implement them, and policy-makers or bioethicists reluctant to approve them, as well as the converse. The purpose of this qualitative research study was to gain a better understanding of perceived risks and benefits of biomedical HIV cure research among these three groups.

## Methods

### Study Setting and Participants

Using a non-probabilistic purposive sampling technique, we conducted 36 key informant interviews from the three groups described above and analyzed the reponses. The first group included 12 interviews with PLWHIV (≥ 18 years, 7 males and 5 females) recruited from a subset of respondents in a social science quantitative survey on willingness to participate in HIV cure research conducted in the United States [3]. None of these individuals were enrolled in biomedical HIV cure research at the time of the interview but were potential volunteers for or had previously volunteered in such research. The second group included 11 interviews with biomedical researchers involved in HIV cure research. These individuals represented eight academic centers in the United States conducting biomedical HIV cure research and various HIV cure research modalities. The final group included 13 interviews with policy-makers or bioethicists, representing regulatory agencies and institutional review boards involved in HIV cure research and recruited from HIV cure stakeholder lists. We maintained a contact log during the study. We contacted 30 patient-paticipants and 12 (40% acceptance rate) accepted an interview, 36 clinician-researchers and 11 (30.6% acceptance rate) accepted an interview and 48 policy-makers/bioethicists and 13 (27.1% acceptance rate) accepted an interview. We did not collect detailed demographic variables from the key informants. Key informant interviews allowed us to solicit in-depth and candid opinions of a broad range of stakeholders effectively [9]. Qualitative inquiry was appropriate to explore the meanings and magnitude of perceived risks and benefits associated with HIV cure research. Further, qualitative research can identify rich narratives and lived experiences not captured in quantitative research and does not make assumptions about a priori HIV cure research literacy of respondents. The type of key informant (e.g. PLWHIV, clinician-researchers, and policy-maker/bioethicist) formed the unit of analysis and served as the key identifier allowing us to compare the perspectives of the three types of informants. HIV cure community advisory board members served as study co-investigators and were involved in the study design and review of data.

### Data Collection

A public health professional (study principal investigator) trained in qualitative research methods undertook the in-depth interviews with key informants (lasting between 30 and 75 minutes) via telephone or in person from September 2015 –January 2016. Her previous experience as a manager of an HIV cure research collaboratory provided a unique opportunity to identify key informants and collect meaningful data. All interviews were audio-recorded, except for one participant who declined recording but accepted note-taking. All interviews took place in English. The standard interview guide included questions regarding perceived risks and benefits of HIV cure studies, including perceptions of what would constitute too much risk (**Table 1**). Key informants were provided an IRB-approved project fact sheet explaining the study objectives and understood that the interviewer was a doctoral candidate interested in formative research on perceptions of risks and benefits of HIV cure research in the United States in collaboration with HIV cure community advisory board members For the most part, respondents were asked to generate free form the types of HIV cure studies they thought were risky. We conducted interviews until we achieved data saturation and a balance in the number of key informants across three categories.

**Table 1:** Questions from IRB-Approved Key Informant Interview Guides.

**Interview Guide for Patient-Participants**
**Introduction**
• Can you please tell us more about your history of participating in HIV research?
• Have you participated in HIV cure research? (If so, probe for details.)
**Risks and Benefits**
• What benefits do you think there are to participate in HIV cure studies?
• What risks do you think are there to participate in HIV cure studies?
• What do you think would be “too much risk” in HIV cure studies?
• Are there studies that you would not participate in? Why?
• What do you consider the safest HIV cure research method? Can you please tell us why?
• What do you consider the riskiest HIV cure research method? Can you please tell us why?
**Interview Guide for Clinician-Researchers**
**Introduction**
• Can you please tell us more about your role in implementing HIV cure research?
**Risks and Benefits**
• Why do you think your patients/participants want to join HIV cure research?
• Are there studies that you would not recommend your patients/participants to participate in? If so, what are they?
• Do your patients/participants incur risks while participating in HIV cure studies? If so, which ones?
• What do you think would constitute “too much risk” in HIV cure studies?
• What do you consider the safest HIV cure research method? Why?
• What do you consider the riskiest HIV cure research method? Why?
**Interview Guide for Policy-Makers or Bioethicists**
**Introduction**
• Can you please tell us more about your role in HIV cure research?
**Risks and Benefits**
• Do you think there are benefits to participate in HIV cure studies? If so, what are they?
• What do you consider the safest HIV cure research method? Why?
• What are some of the risks to participate in HIV cure studies?
• What do you think would constitute “too much risk” in HIV cure studies?
• Are there studies that you would not approve?
• What do you consider the riskiest HIV cure research modality? Why?

### Ethics Statement

The Non-Biomedical Institutional Review Board (IRB) of the University of North Carolina at Chapel Hill approved the study (study #14–2672). Key informants provided oral informed consent since interviews were conducted telephonically (with the exception of one, conducted in person). All key informants received a copy of the informed consent form before the interview. Oral consent was IRB-approved and documented on an individual participant worksheet. Measures were taken to protect the confidentiality of all study participants, including conducting the interviews in a private space and de-identifying all study-related documents and transcripts.

### Data Analysis

We transcribed the recorded interviews verbatim. One researcher reviewed the transcripts for completeness and accuracy by vetting the audio recordings against the transcripts. To protect informants’ identities, we redacted all personal identifiers from the transcripts. We did not return transcripts to participants for comment or correction. Participants did not provide feedback on the findings, although HIV cure community advisory board members well-versed in HIV cure research assisted with the interpretation of the data. We used a combination of grounded theory (to understand the realities anchored in the view of the key informants) and phenomenology (to capture the essence of a phenomenon and the lived experiences of individuals) [10] as our methodological approaches. We performed thematic analysis using a priori codes and data-driven, emergent codes. Main techniques for identifying codes and sub-codes included repetitions, use of transitions, similarities and differences, omissions and pauses, metaphors, analogies, specificity, emotions and extensiveness of coverage [11][12]. The codebook contained the code name, brief description and examples. One researcher applied the thematic codes to the data, and a research assistant subsequently examined the coded texts for accuracy. Discrepancies were resolved via discussions and consensus to reach validity and consistency in interpretation of the data. We used MAXDQA (version 12.1.3, Berlin, Germany) for analysis.

## Results

Our qualitative data revealed four main themes. 1) Potential HIV cure study volunteers noted needing more information and education about the potential risks of HIV cure research. 2) Clinician-researchers, policy-makers and bioethicists showed less awareness of social and financial risks of HIV cure research than PLWHIV. 3) Most respondents across the different categories of informants identified some risks that were too great to be acceptable in HIV cure research, although a subset of PLWHIV did not place an upper limit on acceptable risk in the search for a cure. 4) PLWHIV showed a greater awareness of potential psychological benefits of participating in HIV cure research than other groups of stakeholders.

### Perceived Risks of HIV Cure Research

We asked PLWHIV, clinician-researchers, and policy-makers/bioethicists to discuss perceived risks related to HIV cure research. The majority of PLWHIV focused on potential social risks of HIV cure research, as well as psychological, emotional, and financial risks, and admitted needing basic information regarding possible clinical risks. Clinician-researchers had the most in-depth understanding of clinical risks related to HIV cure research. Policy-makers/bioethicists named possible HIV cure research risks; however, they expressed more concern with risk categories such as known versus unknown risks, short-term versus long-term risks, and real versus theoretical risks.

Some of the PLWHIV were unable to name specific clinical risks related to HIV cure research. For example, one participant said that s/he “[didn’t] think there would be any risks.” Another respondent said that the field of HIV cure research presents a “brave new world,” and admitted that there were many clinical risks but could not name any specific risk. Another key informant thought that the margin of risk was low for HIV cure research since there were many strategies being investigated at present.

PLWHIV, compared to other key informants, focused more explicitly on the topic of physical pain, either associated with investigational interventions and their side effects (such as stem cell transplants and their adverse effects) or association with study procedures or biopsies (such as spinal taps and bone marrow biopsies). Pain was a subjective perceived clinical risk from the perspective of PLWHIV. Further, several conversations touched upon individual experience with HIV treatment, HIV-related complications and the presence of co-morbidities that could present additional risks. The two most often cited perceived clinical risks by PLWHIV were developing resistance to antiretroviral treatment and the risk of developing cancer as a result of being in a study. Additional clinical risks pertained to study procedures as well as possible increase in viral loads, decrease in CD4 counts, opportunistic infections, developing AIDS, worsening of co-morbidities, becoming sick, nausea, hair loss, and dementia associated with reactivation of the HIV reservoir in the brain. Several key informants living with HIV expressed wanting to live long, healthy lives, like their HIV-negative counterparts. Finally, uncertainty about possible unknown risks would be a deterrent to participation. One respondent living with HIV said that the possibility of “cure failure” would prevent him/her from participating in cure research. **Table 2** summarizes perceived risks of HIV cure research from the sub-sample of PLWHIV.

**Table 2:** Perceived Risks of HIV Cure Research from People Living with HIV (PLWHIV), n = 12, United States (2015–2016).

Themes	Quotations
**Clinical or Medical Risks**	***“****My risk is more personal*. *Am I going to get into a cure study were my HIV could go ballistic on me and possibly wipe me out*. *So for me*, *it’s more personal but there are definite risks out there”*
	*“Any stem cell transplant research may have some risks or cancer drugs like the HDACs and shock and kill drugs may have some side effects”*
Resistance to ARVs	*“For me*, *the biggest risk is… I am going to look at the potential for shortening my own life*. *I have very young children so this matters to me*. *If something were really risky and I became resistant to medication… there is a nervousness about that”*
Cancer	*“Cancer*, *irreversible cellular damage and untreatable medical consequences as a result of the study”*
Risks Related to Procedures	*“Any type of surgical procedures or quite painful testing*. *[I am] too old for additional surgeries”*
	*“Just mainly the side effects from all the procedures”*
Pain	*“Procedures that are painful such as biopsy”*
	*“Physical pain”*
	*“I would need detailed explanation of all invasive procedures*, *including those that may be painful”*
	*“Would prefer to have pain kept to a minimum*. *If bone marrow/spinal taps are regular operations*, *I prefer to be under anesthesia (completely unconscious)*. *I do have a needle phobia that has gotten better with time and number of blood draws*, *but [I] do not like to look at needles”*
Permanent or Irreversible Harms	*“Toxicities and long term side effects”*
	*“Permanent damage to my organs or health”*
	*“Fear of being damaged (…) discourage me from biopsies”*
Debilitation or Death	*“Risk of life or debilitation”*
	*“Possibility of death”*
	*“If I know that I will die from doing this*, *because I want to live a longer life just like everyone”*
	*“Other than my death*, *I cannot imagine any potential risk that would discourage me from helping find a cure for HIV”*
Scientific Uncertainty	*“How does it work*, *what can go wrong and what can go right? What is my risk exposure? Basically*, *the more I understand*, *the more I'm ok with it*. *The more that is unknown or fuzzy*, *the less comfortable I'd be”*
**Social Risks**	*“There are risks to some people socially*. *(…) Sometimes when I travel and someone sits down on a plane*. *And the first thing people ask you is “what do you do for a living” and I say that I am a patient advocate and this opens a whole can of worms as far as my hemophilia and HIV and the whole nine yards”*
Poor Treatment by Research Staff	*“Clinical staff that [who] take my time for granted*. *I have been HIV+ since 1988*. *Many staff assume [that] you do not work nor have a career*.*”*
Transmitting HIV to Others	*“I would never put my husband at risk*. *He takes Truvada as a pre-prophylactic*. *We use condoms every time and I have an undetectable viral load and am very compliant with taking all medications”*
	*“Passing it to someone else”*
Disclosure (or breach of confidentiality)	*“If it becomes obvious to the outside world that I have HIV/AIDS then it would be a challenge*. *I am discreet in whom I tell”*
	*“Fear that personal info won't be protected properly and could fall into hands that use the information for nefarious reasons”*
	*“HIPAA not being adhered to”*
Unwanted Media Attention	*“Media attention”*
	*“Publicity of my status”*
	*“Inappropriate media coverage or publication that reveals private or identifiable information”*
Identity Risks	*“22 years is a long time and I went back to school after learning that I was HIV positive*. *My identity is connected to have HIV*. *(…) I never thought about a cure changing who I am”*
	*“Have thought a lot about "losing my HIV+ identity"*. *It is complex*, *ya know?”*
Losing Employment	*“Any risk of losing my career or the risk of infecting others would be a total discouragement to participate”*
	*“I am at the end of my working career (less than 4 years [left])*, *and would not want to experience side-effects that would compromise my ability to work”*
Losing Access to Loved Ones	*“If I had to stay at a clinic for more than one overnight stay without seeing my fiancé*.* *.* *. *As long as we have access to each other be it FaceTime or in person on a daily basis I am good”*
Stigma	*“[Fear of] stigma”*
**Financial Risks**	*“There are some risks–including ability to maintain disability insurance*. *If you [have] been on disability for several years*, *then the insurance companies can say you have to go back to work*. *For me*, *it would be difficult to go back to work*. *There is the financial risk”*
	*“There is of course financial risk*. *There is the risk versus reward*. *It does take time out of your life”*

Clinician-researchers had expert knowledge about possible HIV cure research clinical risks (**Table 3**). The two HIV cure research modalities that were associated with the most perceived clinical risks were latency-reversing agents and stem cell transplant/gene therapy. In general, clinician-researchers were adamant about the imperative to reduce clinical risks whenever possible. They cautioned that scientists need to be careful in interpreting risk information, such as side effects of the investigational compounds, especially outside of the context of diseases for which they have been previously approved. For example, latency-reversing agents were developed as chemotherapy to treat advanced cancers or malignancies, not treatable chronic conditions. Therefore, the assessment of potential risks and/or toxicities is very different between cancer and HIV. For example, side effects that would be tolerated by oncology doctors might be unacceptable for HIV clinicians. A biomedical researcher provided the example of the FDA’s decision to place a panobinostat (cancer drug) study on clinical hold in 2015. The main concern is that “if you intervene with a potentially toxic drug with unknown benefits with respect to cure, [and] if these drugs have irreversible side effects, then you have induced harm in someone without really providing them with any direct benefit.”

**Table 3:** Summary of Perceived Risks of HIV Cure Research from Clinician/Biomedical Researchers, n = 11, United States (2015–2016).

**Perceived General Risks**
• Risk of procedures; phlebotomies, leukaphereses, invasive biopsies
• Risk of pain or discomfort
• Risk that interventions will have unanticipated immediate or delayed toxicities with greater impact to health
**Perceived Risks Specific to Latency-Reversing Agents**
• Recorded adverse events (AEs)–mild to moderate on the clinical trial scale
• Gastro-intestinal side effects, nausea
• Fatigue
• Vomiting
• Anemia
• Toxicities (bone marrow suppression, myalgia, dysphoria)
• Long-term toxicities (mutagenicity, carcinogenicity)
**Perceived Risks Specific to Stem Cell Transplants/Gene Therapy**
• Risks of infection/contamination
• In the longer-term, could do something genetically to cells that will make them more susceptible to give rise to cancer
**Perceived Risks Specific to Checkpoint Blockers**
• Chronic inflammation on the immune system

Overall, clinician-researchers mentioned the need to stay vigilant regarding emerging data about risks in early-phase HIV cure studies. They stated that the history of medical research has taught us that some toxicities simply cannot be detected in pre-clinical experimental models, and also referred to the incremental nature of scientific discovery. In the gene therapy world, for instance, “It’s a scary thing to be manipulating DNA and one has to be very aware of the possibility for harm there. [But] as much work has gone into it, it would appear to be safe, otherwise it would not have moved on into cure trials.” Besides potential clinical risks, clinician-researchers also referred to possible opportunity risks of joining clinical studies. This would mean that if a PLWHIV volunteers in a study, s/he may not be able to participate in a subsequent study. Some of them also asked for more nuance when discussing risks related to HIV cure research, because each intervention type, or even each study, should be assessed individually. Clinician-researchers emphasized that different HIV cure research strategies had varying levels of risks, some of which remain unknown.

Comparably, policy-makers/bioethicists identified possible HIV cure research risks, but their approach emphasized risk categories. Policy-makers/bioethicists displayed more risk aversion than other groups of stakeholders and showed concerns with types of risks, such as known versus unknown risks, short-term versus long-term risks, and real versus theoretical risks. For example, a bioethicist, discussing scientific uncertainty, said: “Well, it’s the risk of the unknown… right?”. Policy-makers/bioethicists recognized the difficult nature of assessing risks in HIV cure research, and contrasted this to the HIV treatment field where drugs have well-quantified risks associated with them. A regulator said that “[We are making] apples to oranges comparisons at times, even within the same modality.” A bioethicist recognized that clinical studies are not designed for the best medical interest of the individual patient, but to answer a specific research question that will lead to generalizable knowledge. Further, another policy-maker pointed out that the availability of potent HIV treatment options and the relatively healthy status of virally suppressed individuals raise the safety threshold that must be considered in HIV cure research. **Table 4** summarizes the perceived risks of HIV cure research from the cross-section of policy-makers/bioethicists.

**Table 4:** Summary of Perceived Risks of HIV Cure Research from Policy-Makers/Bioethicists, n = 13, United States (2015–2016).

**General**
• Various toxicities and side effects, known and unknown
Risks of immediate/short-term, chronic or delayed/long-term morbidity or mortality (e.g. cancer)
• Risks related to the specific intervention and/or agent
• Procedure or monitoring-related risks
• Risks associated with treatment interruption
Risk of viral rebound or reactivation of disease
Potential health risks (e.g., reduced T cell levels) if the virus is allowed to replicate freely for an extended period of time
Change in the phenotype of the virus
Developing resistance to antiretroviral treatment
Transmitting the virus to partners
• Development of resistance to antiretroviral treatment
• Limited treatment options in the future
• Permanent harm of the intervention being used
• Relative risks (e.g. agent/intervention versus treatment interruption)
• Theoretical risks (e.g. possibility of death)
**Latency-Reversing Agents**
• Toxicity risks of the specific agent
• Possible long-term consequences of reactivating latent virus
• Risk of stirring up other potential latent retroviruses or reactivating other viruses (such as Herpes Simplex Virus)
**Stem Cell Transplants/Gene Therapy**
• Risks associated with chemotherapy and/or conditioning to ablate the immune system

Narratives of PLWHIV included potential social risks related to HIV cure research. Examples of potential social risks included: possible poor treatment by research staff, transmitting HIV to others, disclosure, unwanted media attention, losing one’s identify as being HIV-positive, losing employment, losing access to loved ones, and stigma. Some PLWHIV were concerned that they could be treated poorly or taken for granted by clinical researchers or nursing staff who might assume that they did not have jobs or other time commitments. The risk of transmitting HIV to others in the course of HIV cure research experimentation and unsuspected viral rebound were cited as concerns. Another perceived social risk was inadvertent disclosure of HIV status that would lead to distrust in the community of people living with HIV. A subset of informants feared that their personal information would not be protected properly. Furthermore, unwanted media attention or publicity regarding one’s HIV status were perceived as serious personal and social risks of study participation. A minority of informants mentioned the risks associated with losing one’s identity as someone living with HIV, although the majority answered the question in the reverse, saying that they “would gladly lose [their] HIV-positive identity for a cure.” Other social risks included losing employment or losing access to loved ones in the course of study participation. Besides social risks, informants perceived financial risks related to study participation. Examples included concerns around maintaining disability insurance or income, including private or Social Security, current health care or insurance coverage. **Table 2**(above) contains perceived social and financial risks from the sub-sample of PLWHIV.

Stem cell transplantation, gene therapy, latency-reversings agents, and combinatorial approaches were perceived to be the riskiest HIV cure research strategies by clinician-researchers and policy-makers/bioethicists. There was little comparable data from PLWHIV given their limited medical and scientific knowledge about the clinical risks of HIV cure research. Clinician-researchers and policy-makers/bioethicists stated that stem cell transplants and gene therapy were the perceived riskiest approaches, given the comparatively higher mortality associated with allogeneic transplants. Further, some clinician-researchers and policy-makers/bioethicists reported that bone marrow transplants for patients who did not have cancer were considered extremely risky because of the conditioning procedures and the receipt of genetically modified cells. Relative to other HIV cure research strategies, however, stem cell transplants were associated with the largest reduction in the size of the HIV reservoir to date, and thus were perceived to have the highest chance of succeeding in chronically infected patients. Zinc finger nucleases were one type of gene therapy that was categorized as being risky, especially when combined with treatment interruption. Some informants perceived any interference with the human genome as unnerving. Additionally, latency-reversing compounds were perceived to be risky because they attempt to reactivate quiescent virus. These agents are borrowed from oncology and may have unpleasant side effects; and they were perceived to not have yet been associated with a substantial reduction in the size of the replication-competent HIV proviral DNA reservoir. While the cancer drugs have led to transient increases in cell-associated HIV RNA, they were perceived to target important host enzymes and processes and may act in ways that could cause secondary malignancies.

In contrast, the HIV cure research strategies perceived to be the safest by clinician-researchers and policy-makers/bioethicists were early ART, vaccinations/immune-based strategies, monoclonal antibodies, and reservoir assessments. There was little comparable data that could be analyzed from PLWHIV. Early ART was considered safest and most logical by key informants because the drugs are already FDA-approved and have proven efficacy to treat (not cure) HIV. Vaccinations or immune-based therapies were also considered safe, particularly those utilizing *ex vivo* expanded autologous cell systems because they do not introduce foreign agents in the body. Monoclonal antibodies also received the safety vote by most clinician-researchers and policy-makers/bioethicists. Finally, reservoir assessments were also considered safe since they were observational and did not require administration of foreign agents.

### Perceptions of Unacceptable Risks in HIV Cure Research

All three categories of informants described what would constitute too much risk in HIV cure research. A subset of PLWHIV did not place an upper limit on acceptable risk. **Table 5** summarizes perceived unacceptable risks.

**Table 5:** Perceived Unacceptable Risks in HIV Cure Research from Key Informants (People Living with HIV, Clinician-Researchers and Policy-Makers/Bioethicists, n = 36), United States (2015–2016).

Themes	Endorsements	Quotations
**Regulations and Clinical Holds**	Policy-Maker/Bioethicist	*“There is a category of too much risk in HIV cure studies*. *We have put protocols on clinical hold because we thought they were too much risk*. *Either due to previous use of the drugs or strong biological plausibility of (…) severe adverse reactions*. *A lot of this is a judgment call*. *For example*, *how frequent it is likely to occur*. *If [it’s] likely to occur in more than 2–3% of patients and [if it would be] very serious*, *[it] would likely be too much risk”*
	Policy-Maker/Bioethicist	*“This is hard to answer*. *There are treatment modalities that are reserved for metastatic cancer*. *There are a lot of black boxes*. *For otherwise healthy people who have a long life expectancy*, *this may be too much risk”*
	Policy-Maker/Bioethicist	*“Basically*, *under the regulations*, *there are two possible scenarios*. *Insufficient information to assess risk–either not enough information about animal model studies or from previous clinical research to be able to have a good assessment of the safety and dose and duration of the product*. *The other one in the regulation is when we think that the risks are unreasonable*, *when the potential benefits do not outweigh the potential risks*. *These are the rules that we use when we review study protocols”*
	Policy-Maker/Bioethicist	*“I would consider “too much” risk if the study drug or procedure is known to be significantly toxic*. *It is particularly concerning if there are limited data to support or indicate that the drug have the desired effect such as reducing the reservoir*. *Another concern would be if the study failed to include a well thought-of design such as well-defined endpoints. (..) If you’re going to subject people to risky interventions*, *[you] need at least interpretable results to be able to advance the field”*
**Perspectives of People Living with HIV**		
First-in-Human Studies	People Living with HIV	*“Trying an approach never tested on humans or with less than a 60% positive result*. *That said*, *I must feel confident researchers care about my heath*, *first and foremost*, *not lab results”*
		*“Any first in human study without an underlying proof of concept in an animal model”*
Becoming Detectable or Viral Load Increase	People Living with HIV	*“Becoming detectable at extraordinarily high numbers”*
		*“If I were to become detectable again with no recourse”*
		*“My viral load being too high”*
		*“If my dormant virus multiplied in a vengeful way”*
		*“Huge viral rebound”*
Significant Changes in CD4 Count	People Living with HIV	*“If my CD4 count decreases too much”*
		*“Low CD4 count*.* *.* *.*less than 500”*
		*“If my CD4 count (…) pushed me back into the category of having AIDS”*
		*“If the cure research put me back under 200 T cells and I was in danger for PCP pneumonia again*, *that would be too much risk for me”*
Physical Pain	People Living with HIV	*“Well*, *I am really averse to pain*. *If you ask me to go through a bone marrow*, *I’ll run the other way”*
		*“There would need something to keep me from experiencing the pain”*
Cancer Risks	People Living with HIV	*“Cancer risk”*
		*“The overall risk of developing cancer”*
		*“A cure-related case study that is known to cause various cancers of any kind”*
		*“Gene manipulations that result in cancers probably would be the most frightening and unbearable*, *but frankly if I was really sick*, *I would risk a lot more to get better”*
Permanent, Irreversible Side Effects	People Living with HIV	*“Permanent debilitating side effects “*
		*“A risk that would have me end up in irreversibly poorer health than I am now”*
		*“Anything that might become permanent or irrevocable”*
		*“Irreversible damage”*
Hospitalization	People Living with HIV	*“Extended hospital stay”*
		*“Any recurrence of adverse effects from antiviral drugs taken in the past; at least one of which required hospitalization”*
Debilitation	People Living with HIV	*“Too much risk would be an outcome that leaves me disabled without the resources to provide for myself”*
		*“Anything that would cause functional disability such as loss of sight*, *stroke*, *heart attack”*
Death	People Living with HIV	*“Certain death”*
		*“A study where there have been deaths due to the medications prescribed”*
		*“Greater than 1% risk of death”*
		*“Death–I'm a mom to a 5 year old little boy and I want him to have a little brother or sister soon”*
		*“Anything that would kill me–been living with AIDS for twenty [years] and feel I still have left many good years”*

Policy-makers/bioethicists described their primary responsibility as the evaluation of first-in-human protocols. If the FDA considers a protocol unacceptable, the agency issues a set of recommendations to the clinical investigator(s) and may place a clinical hold on any protocol. The assessment is usually based on the available evidence or the strong biological plausibility of severe adverse drug reactions, but this is sometimes based more on judgment than evidence alone. A policy-maker clarified that if severe adverse drug reactions would be expected in more than 2–3% of participants, this would be too much risk. Further, for metastatic cancer drugs repurposed for cure research in otherwise healthy volunteers, the policy-makers interviewed emphasized that there needs to be a clear rationale for moving a specific dose of compound forward.

Policy-makers/bioethicists referred back to the U.S. federal regulations. There are two possible scenarios that would constitute too much risk for them: 1) insufficient information to assess risk–either insufficient data from animal/pre-clinical studies to make a good assessment about safety, optimal dose or duration of product, or 2) insufficient benefits to outweigh the risks. Furthermore, risks would be deemed unacceptable if the procedure or drug was known to be significantly toxic and there would be no counter-balancing procedure to reduce risk, or if a drug was known to be toxic without strong evidence that it would deplete the HIV reservoir. According to some of the policy-makers/bioethicists interviewed, one should be concerned if the study failed to include a well-reasoned rationale for the study design, with insightful endpoints, and with the prospect of interpretable results that would advance the field.

Narratives of PLWHIV yielded variability in the responses and depended on each informant’s risk threshold. For one key informant living with HIV, any first-in-human study that did not have sufficient pre-clinical animal evidence for potential safety and efficacy was considered unacceptable. Other potential volunteers pointed out specific clinical risk thresholds that would be unacceptable, such as those related to analytical treatment interruptions, including increases in viral load above a pre-specified level. Correspondingly, a decrease in CD4 count below a specific threshold would also be unacceptable. Furthermore, some PLWHIV admitted being averse to invasive procedures (such as spinal taps and bone marrow biopsies) and physical pain. Other perceived unacceptable risks were cancer, permanent or irreversible side-effects, hospitalization, debilitation, or death.

The two most-commonly cited unacceptable HIV cure research strategies among key informants were stem cell transplants and anti-PD1 interventions, although there was variability in the responses given, since one of the PLWHIV interviewed had undergone an allogeneic stem cell transplant. A number of clinician-researchers felt that stem cell transplants in otherwise healthy volunteers who were stable and suppressed on antiretroviral therapy would be going too far, especially as scientists were not yet sure what exactly cured Timothy Brown. A clinician-researcher who performs stem cell transplants stated that treatment interruption during the transplantation is perceived as too risky, and that ART should be maintained during the transplantation since there is no benefit of stopping ART and the possibility of engraftment will also be enhanced. Concerning anti-PD1 approaches, a clinician-researcher stated that they have shown significant toxicities in non-human primates and some studies have ceased in humans. With regards to latency-reversing agents, a clinician-researcher cautioned about the risks of causing global activation of T cells, as occurred in an early study in Europe. (The referred study tested immuno-activation with anti-CD3 and recombinant human IL-2 in PLWHIV on ART [13]. The regimen resulted in profound T cell activation and serious side effects, including fever, headache, nausea, diarrhea as well as renal failures and seizures in one of the study participants. The OK3/IL-2 cocktail was found to be too toxic and provided a “lesson learned” for the HIV cure research field in that HIV reservoirs needed to be reactivated without causing global activation of T cells.) Further, a biomedical researcher commented that anything suggesting an irreversible and systemic side effect would be unacceptable; however, it may not be possible to know whether an intervention carries the potential for irreversible and/or systemic side effects until it is tested in humans.

Treatment interruptions indicated in some HIV cure research protocols, and their associated risks, were deemed unacceptable for a subset of key informants across the three categories. A clinician-researcher explained that testing latency-reversing agents with treatment interruptions would be reckless at this point, since the compounds have not yet been associated with a substantial reduction in the size of the replication-competent HIV DNA reservoir, and therefore viral rebound will be almost certain and automatic. Reasons given by PLWHIV for perceiving treatment interruptions as unacceptable included: current low CD4 count and almost guaranteed viral rebound, possibility of losing undetectable status, fear of transmitting HIV to others, and developing resistance to drugs. Since viral rebound is unpredictable, it was perceived as being too risky for some, or associated with risk of increasing viral reservoirs in the body, or even the possibility of death.

Most PLWHIV touched upon potential social and financial risks that would be unacceptable. These included significant changes in quality of life, such as not being able to exercise, walk or speak, increased fatigue, and lack of normalcy. A subset of respondents indicated that becoming detectable for HIV and increased risk of passing HIV to sexual partners would be unacceptable. Other social risks included inability to work or care for family. Unacceptable financial risks were also identified such as insufficient compensation for the required biopsies and interventions or to offset time off work.

Interestingly, three (out of 12) PLWHIV acknowledged that they would not place any upper threshold on risks in HIV cure research. They said that they could not think of anything that would be too much risk and that they would undergo stem cell transplants or ingest latency-reversing drugs. They expressed that they would be “willing to do whatever it takes.” Further, some PLWHIV answered that they perceived no risks of HIV cure research. One key informant said that s/he “[did] not have enough knowledge/information about potential risks to make [an] informed comment.” Another respondent stated that “finding a cure outweighs the risks.” Yet another person indicated that “All I see is benefits in the search for a cure.” This raises ethical issues that will be explored in the discussion section, particularly related to informed consent.

Clinician-researchers expressed the importance of case-by-case analyses to assess risks for individual participants. For example, one said that “I think this is relative to the patient, so if they are very ill, then I don’t think/know that there would be a definition of too much risk for them. But if they are very healthy (…) and their therapy is working and they are suppressed, then these would be the ideal patients to be in cure studies.” Similarly, a clinician-researcher stated that “there are variations from patient to patient. Not all investigators are created the same and not all patients are [either]. And some patients can handle the anxiety of the treatment interruptions better than others. Some might not be good candidates for (…) the gene therapy trials (…) certainly different trials appeal to different types of patients.” Most clinician-researchers recognized the need to respect the autonomy of study candidates in decision-making.

In turn, policy-makers/bioethicists discussed the subjective nature of unacceptable risks. For example, a policy-maker mentioned that “[This is][r]eally gonna depend on the nature of the study, the benefits and the science, [and] how the risks are being managed and controlled.” Similarly, another policy-maker said: “Decision making is not entirely rational. We do not have a rational process to evaluate the risks/benefits for these interventions and a way to decide on the ethical questions. [These] [q]uestions have not yet been solved.” Similar discussions occurred with other policy-makers and bioethicists regarding risk thresholds, who stated that the current availability of potent and safe antiretroviral treatment increases acceptable safety thresholds for HIV cure study participants. Further, the theme of scientific uncertainty emerged with bioethicists who mentioned that several risks of HIV cure research remain unknown. Overall, policy-makers/bioethicists and clinician-researchers generally agreed that limits had to be placed to ensure participant safety and preserve trust in research.

### Perceived Benefits of HIV Cure Research

We assessed perceptions of benefits in HIV cure research. The most commonly-cited perceived benefits by PLWHIV (not enrolled in biomedical HIV cure research at the time of the interview) were socio-emotional in nature.

Across the three groups of key informants, the main perceived benefit of HIV cure research participation related to the social benefit of advancing scientific knowledge and helping future generations. A few clinician-researchers recognized that HIV cure research might have applications to other diseases or conditions. Most clinician-researchers and policy-makers/bioethicists stated that one would anticipate no direct (clinical) benefit to the individual participants in early translational HIV cure research. A number of clinician-researchers mentioned that if there were any benefits, they would be indirect, such as engagement with the research team or screening tests that may identify health issues. Interestingly, a clinician-researcher reported possible isolated clinical benefits of HIV cure research participation. The cited example was a study that resulted in increased CD4 T lymphocyte cells in participants. While HIV was not completely gone from study participants at the end of the study, their body was able to control HIV better. The clinician-researcher described this as “unexpected clinical benefits” and explained that these may demonstrate a missing element in HIV cure research, namely that clinical benefits short of a cure for HIV may emerge.

In the group of PLWHIV, the most prominent perceived personal benefits of HIV cure research participation were the psychological and emotional benefits of contributing to finding a cure. Most PLWHIV felt that psychological benefits should not be discounted as they lead to overall improvements in quality of life, as well as removal from isolation after a difficult diagnosis. Some of the key informants living with HIV said it was “the right thing to do” to participate in HIV cure research and felt pride to be able to be a part of it. A participant who underwent an allogeneic stem cell transplant expressed that he felt tremendous emotional benefits after helping to further medical knowledge. Further, some PLWHIV valued their experience in a prior HIV clinical study and these benefits had little or nothing to do with the investigational intervention itself. There were the psychological benefits of being in regular contact with clinical staff, of being treated with dignity and respect and feeling valued because of research participation. Key informants also expressed a sense of duty, the need to give back and help others, satisfaction in being pioneers, and feeling empowered about their condition.

An unexpected personal benefit of study participation that emerged from speaking with PLWHIV was acquiring information about HIV and being able to educate their peers. Some PLWHIV saw benefits from learning about cutting-edge HIV research, and felt that this information could bolster their advocacy work. Armed with this information, participants felt that they could refer peers to HIV cure studies. Additional potential personal benefits to participation were reported, including getting support from others and being more hopeful. Some of the perceived benefits to study participants were actual ethical requirements of clinical studies, such as information about risks and confidentiality. Another perceived social benefit of participating in HIV cure research identified by PLWHIV was contributing to reducing stigma around the disease. Some of the PLWHIV mentioned that one of the benefits of their participation in HIV cure research would be ensuring that under-represented populations get included in studies. The simple fact of knowing that their groups were included in the research would be sufficient to confer a benefit because people know their own group is being represented. Other benefits included “satisfying curiosity,” “leaving a legacy” and “having a second chance at life.” **Table 6** summarizes the perceived benefits of HIV cure research.

**Table 6:** Perceived Benefits of HIV Cure Research from Key Informants (People Living with HIV, Clinician-Researchers and Policy-Makers/Bioethicists, n = 36), United States (2015–2016).

Themes		Endorsements	Quotations
**No Expectations of Direct Benefits**		Policy-Maker/Bioethicist	*“There are no benefits in terms of changing the HIV disease course for that particular person*. *I doubt that any of these will lead to HIV cure or benefits in terms of how they are treated at this point in time*. *If there are any benefits*, *they could be more indirect from engagement with medical staff or getting extra labs that they would not have gotten otherwise*. *[It’s] [v]ery unlikely that there will be any direct benefits”*
		Policy-Maker/Bioethicist	*“There might be some indirect benefits for these early trials*. *Proof-of-concept studies and types of modalities carry considerable amount of risks*. *There are no direct benefits [and we] need to manage expectations*.*”*
		Policy-Maker/Bioethicist	*“At this early*, *exploratory stage of development*, *there can be no anticipation of direct benefit to participants*. *There is the potential for a general*, *societal benefit of furthering scientific knowledge”*
**Societal Benefits:**			
	Advancing Scientific Knowledge	People Living with HIV	*“I think all knowledge is important and the only way that we learn in this world is through failure so I do feel that early clinical trials now (…) What has always motivated me is that every bit of information can help somebody else”*
			*“Being part of ground breaking research is huge for me*!*”*
			*“Increasing the common good and helping to advance knowledge”*
			*“The most important part of joining any study for me is the chance to help forward the science and knowledge*.* *.* *. *The hope for future generations”*
**Personal Benefits:**			
	Psychological Benefits	Person Living with HIV	*“The benefits to me were emotional knowing and believing that I helped further medical knowledge*. *In simplistic terms*, *the researcher told me that the virus returned and we stayed in touch*. *The researcher said to me*: *“Do not underestimate… you have had a profound impact on medical research because we have found information that we would not have been able to know before*. *And more information than just simplistically about this particular scenario*.*(*.* *.* *.*) I felt and still feel very satisfied that I participated and did that”*
	Intermediate Successes	Clinician-Researcher	*“The Sangamo-type trials*, *there is a reasonable expectation that they might give a benefit to the participant’s baseline immune function (…) The HIV may not be completely gone*, *but the therapy has helped the body control it better (…) And this is a bit of an aside*, *but I think in the field of course [what] you worry about is how long it will take all of us to succeed and we really don’t know*. *You hope that there will be intermediate successes along the way”*
	Information and Education	Person Living with HIV	*“I think that the benefit is education*. *We are not educated enough*. *And even though this thing has been around for years and years*, *we still need the education*. *There is not a lot of knowledge*. *There is a lot of stigma because there is a lot of ignorance*. *Because people don’t know*. *Even among doctors”*

## Discussion

Our findings provide unique insight into the perspectives and preferences of various stakeholders regarding HIV cure research in the United States. The study extends the literature by focusing on HIV cure research perceptions in the United States, including PLWHIV, clinician-researchers, and policy-makers/bioethicists’ perspectives, as similar stakeholder perception studies have been published in other countries [14][15][16]. The qualitative inquiry yielded a rich understanding anchored in the participants’ own categories of meaning and knowledge base. Narratives around perceived risks and benefits of HIV cure research modalities revealed different notions about these concepts among stakeholder types. For example, while clinician-researchers focused on clinical risks of HIV cure research, PLWHIV had a deeper connection with perceived social and financial risks, as well as potential non-clinical benefits. Overall, the results offer guidance about which risks and/or benefits biomedical HIV cure researchers should take into consideration when designing, implementing, and communicating about HIV cure studies and provided a foundation for identifying some of the factors that influence decision-making in HIV cure clinical research.

Risk perceptions play a major role in determining which HIV cure studies receive regulatory and institutional approvals, inclusion and exclusion criteria, design considerations, safety and monitoring protocols, recruitment strategies, informed consent content and processes, and decisions made by PLWHIV on whether or not to put their bodies on the line and participate. Some risks are known while other risks are unknown. This is a major hallmark of first-in-human studies which carry greater scientific uncertainty than later phase trials [17]. Further, PLWHIV may not fully comprehend the risks of HIV cure research participation, or they may overestimate the clinical benefits, resulting in therapeutic misestimation or misconception [18][19]. The discourse around potential social risks strengthens the need to capture and address potential social harms during HIV cure research. Moreover, decisions to participate in HIV cure research are not divorced from the impact of HIV on the daily life of potential study volunteers and their views on currently available treatment options [20]. A case in point was that one of the most commonly cited risks of participation in HIV cure research involving a treatment interruption was that HIV could rebound and become unmanageable. Attending to safety protocols to catch viral rebound early will be paramount for studies that include ARV interruptions.

Our study further highlights the challenges of making risk determinations in HIV cure research, including acceptable and unacceptable risks. The U.S. Code of Federal Regulations mandates that “risks to subjects” be “reasonable in relation to anticipated benefits, if any, to subjects and the importance of the knowledge” to be gained from the research [21]. There is limited concrete guidance for implementing this requirement, however [22]. Further, the notions of “acceptable risk” may vary by stakeholder groups. While the FDA or an IRB may deem an Investigational New Drug (IND) acceptable from a regulatory standpoint, volunteers must still be willing to participate and provide individual informed consent. Clinician-researchers must also assess risks in relation to the individual participant’s medical condition. Inherent scientific uncertainty and value judgements make acceptable risk threshold determinations difficult to ascertain at various stages of HIV cure research. Our findings are consistent with the emerging literature around the risk/benefit ratio challenge in HIV cure research [2]. Understanding as much as possible about actual risks of a particular study or intervention, the margins of what can be known about the risks of a new intervention, along with considerations of the value placed on those risks, will be important for determining what studies should be undertaken. For the majority of key informants, there was clearly a category of too much risk in HIV cure research. Nevertheless, a subset of PLWHIV did not place an upper threshold on acceptable risk. Our findings revealed the heterogeneity of perceptions around unacceptable risks for PLWHIV. We found that the question of too much risk should require constant inquiry as the HIV cure research and bioethics associated with it will continue to evolve. Acceptable risk thresholds will be contingent upon the population being studied, the modality under investigation, the available scientific evidence of safety and efficacy at any given time, and the alternatives to study participation, e.g. potent antiretroviral treatment. Further, most of the ethics literature on HIV cure research has discussed risk acceptability between ‘otherwise healthy volunteers’ on effective HIV antiretroviral treatment as being distinct from late-stage oncology patients asked to participate in novel and risky first-in-human studies [17][23]. Less attention has been paid, however, to other potential helpful medical analogies for the purpose of assessing risks and benefits in HIV cure-related studies, such as studies that would allow ‘otherwise healthy’ recipients of solid organ transplantation to stop taking strong immune-suppressive medications [24][25]. Moreover, our data were sparse in relation to how risk mitigation strategies or provisions for research-related injury affect perceptions of risks and benefits in HIV cure studies[26], and these considerations would need to be expanded in future research.

Knowledge and perceptions around risks of HIV cure studies among PLWHIV may come from multiple sources, including clinician-researchers and informed consent forms at the point of entry into a clinical study. A body of literature has focused on the role of gist knowledge–defined as “qualitative, more general cognitive representation of understanding” [27]. This literature focuses on the role of heuristics–or cognitive shortcuts–in medical decision making, suggesting that trade-offs are negotiated by study participants to reduce the cognitive burden of decision-making, even at the risk of reducing decisional accuracy [27]. Gist knowledge has an influence in decisions to participate in research, often above verbatim knowledge found in informed consent forms. Further, for good or for bad, potential study volunteers may form opinions about HIV cure strategies via other settings, such as the popular/social media, study recruitment materials or advocacy or literacy pieces that contribute to shaping public perceptions around the research [28]. This underscores the need to provide accurate information about actual risks and benefits (or lack thereof) of HIV cure research strategies in all platforms. Similarly, research has found that prospective study participants may have already made a decision as to whether to participate in a clinical study before they come in the door or receive an informed consent form [29]. This shows that the process of informed consent may actually begin before the potential risks, benefits and objectives of a study are clearly explained to a study candidate. HIV cure research implementers should therefore pay attention to perceptions of risks and benefits and the process of recruitment, provided that candidates may have already made a decision to participate before they are consented [29]. Needless to say, the language used in the informed consent form is paramount and there should be conscientious efforts to streamline and increase the readability of informed consent forms [30] and participant’s understanding of the research should be assessed.

Moreover, ethical guidelines remain clear that clinician-researchers have an obligation to protect study participants from unjustified or excessive risks [31]. The placement of limits on permissible risks is warranted by the need to protect the research enterprise and the study participants, sometimes from themselves [31]. Currently, IRBs and regulatory agencies provide the risk determinations of risk permissibility, yet there is no completely objective yardstick with which to assess risks. Our findings are consistent with prior literature assessing perceptions of risks and risk acceptability in gene therapy clinical risks, in that risks are difficult to assess given the uncertainty of clinical trial outcomes in translational research [32]. Deakin and colleagues reminds us that we need to pay attention to clinical context and preclinical evidence, value judgments about risks and benefits, as well as subjective factors that may reflect the individual experience of stakeholders, including involvement in clinical care [32]. Additionally, acceptable risk thresholds can change over time and with the advent of new technologies (e.g. potent antiretroviral drugs). Unquestionably, perceptions of what interventions present too much risk will need to be taken into account when designing and implementing studies in order to earn and maintain basic public confidence and trust in the HIV cure research field [33].

Our qualitative findings show that PLWHIV noted substantial anticipated psychological and emotional benefits from participating in HIV cure research. It is possible that participation in HIV cure research can change the meaning of one’s diagnosis from a traumatic event to a potentially meaningful one. This finding is consistent with what has been found in an in-depth qualitative study of HIV cure stakeholders in North Carolina, USA [34] and with the early-phase cancer literature [35]. The lack of direct anticipated clinical benefits is also well-documented in early phase HIV cure studies [1][17] [36][37], however, our study also revealed possible intermediate successes along the way and the chance of psychological successes.

HIV cure scientists should appreciate the perceived intangible benefits to participation and seriously consider the altruistic appeal to scientific advancement when conducting recruitment efforts. The topic of societal benefit has received some attention in the context of high-risk/low-benefit studies, because it is an important motivator of participation in HIV cure research [20][38]. This is consistent with connected bodies of literature, including HIV prevention research [39]. Social altruism is defined as “individual[s] weighing potential social benefits for research participation over and above any personal risks associated with trial participation” [39]. In social altruism, study participants feel that the societal benefits of study participation outweigh potential personal, health, clinical or social risks [39] and they attach utility to more than the benefits to him/herself [40]. Further, scientists have witnessed a positive relationship between altruism and quality of life in the HIV prevention field, as research participation adds meaning and purpose to the lives of participants[39]. It was found that nurturing a sense of altruism among study participants could facilitate recruitment in studies, and altruism is very important in clinical studies that involve greater personal risks [39]. Nevertheless, in our key informant interviews, the wish to contribute to scientific knowledge around HIV cure was often mixed with desire for personal benefit. The topic of mixed altruism has been under-explored in research around decision-making toward HIV cure clinical studies. While study participants may be motivated to join studies for perceived personal benefits, they can still understand that the overall intent of the endeavor is to gather generalizable scientific knowledge [41]. **Table 7** incorporates possible considerations regarding risks and benefits of HIV cure research.

**Table 7:** Considerations Regarding Managing Risks and Benefits in HIV Cure Research.

**Considerations regarding risks**
• HIV cure clinical investigators have an ethical duty to convey understanding of risks to potential study participants. Risks must be minimized and they must be reasonable in relation to the potential benefit that study participants may realize. Ethical guidelines must continue to protect study participants against unacceptable risks.
• It is important to differentiate the risks that stem from investigational interventions or agents, study visit procedures, and monitoring-related risks, and understand how deviations from standards of care (e.g. analytical treatment interruption) can add to the actual and perceived risks.
• The heterogeneity of HIV cure clinical studies and the scientific uncertainty make the reliability of risk/benefit judgments difficult. Caution should be exercised when evaluating the various HIV cure research strategies. Care must be taken prior to exposing HIV cure study participants to substantial likelihood of serious risks, particularly since PLWHIV have excellent treatment options to maintain health.
• It is important to remember that potential participants may believe that more benefits may be realized than is possible, particularly in early phase studies, which may cause them to be willing to accept more risk exposure. There should be sustained education efforts around HIV cure research and the potential risks for PLWHIV interested in participating in research. Verification of understanding should be implemented as part of the informed consent process.
• More formative research is needed on actual and perceived risks of participating in HIV cure clinical studies, involving collaboration between biomedical researchers, social scientists and other stakeholders including community representatives. Attention should also be devoted to risk communication and evaluating future risks by HIV cure biomedical investigators, such as development of cancer many years after study participation has ended.
• Social harms should be captured as part of HIV cure clinical research participation, and interview protocols should seek to discover if they have occurred.
**Considerations regarding unacceptable risks**
• Perceptions of what constitutes too much risk or unacceptable risks should be taken into account when designing and approving studies, as they influence the ethical development and implementation of HIV cure research.
• There should be safeguards in place to ensure that unnecessary risks are not built into study protocols, and policy-makers/bioethicists have a role to play in creating such policies.
• More empirical research is needed on what represents too much risk in HIV cure research as the field is evolving quickly and there is scientific uncertainty associated with the various modalities under investigation.
**Considerations regarding benefits**
• Researchers have the responsibility to report the associated lack of anticipated clinical benefits in early-phase HIV cure studies.
• Researchers need to appreciate that study participants may perceive and receive tremendous psychosocial and emotional benefits from being in a study.
• Researchers should clearly distinguish between individual and societal benefits in informed consent forms, including potential benefits from the interventions (if any) versus inclusion benefits.
• Compensation should not be presented as a benefit to HIV cure research participation.
• Examinations and study interventions should not be considered guaranteed benefits of HIV cure study participation. Laboratory tests may lead to greater knowledge and understanding of one own’s health status, but sometimes individual research results are not shared with study participants since they do not have immediate clinical relevance.
• HIV cure research participants should know that interventions in Phase I and Phase II clinical trials are experiments that evaluate basic safety and they are meant to generate knowledge for the benefit of society. They should be reminded that only a small minority of early-phase studies lead to effective interventions.
• HIV cure researchers should be reminded that HIV cure research participation relies fundamentally on altruism of study participants.
• More empirical research is needed to understand perceived and actual benefits of participating in HIV cure clinical studies, involving collaboration between biomedical researchers, social scientists and other stakeholders, including community representatives.

### Strengths and Limitations

This study reviewed perceived risks and benefits of HIV cure research among various stakeholders in the United States, using qualitative inquiry. We must acknowledge a number of limitations, however. First, the knowledge base of HIV cure research was limited among PLWHIV. This may have limited their perceptions around potential risks and benefits of such research. PLWHIV were not enrolled in biomedical HIV cure research at the time of the interview and discussed perceptions of hypothetical risks and benefits of research. Nonetheless, this group of informants revealed a need for greater education around HIV cure research strategies and their potential risks. Second, we only interviewed three types of informants and other significant groups were not represented, such as pharmaceutical companies or funders. It is difficult to ascertain bias in our sample. We suspect self-selection and social desirability biases affected the responses about altruistic motivations of PLWHIV versus clinician-researchers and policy-makers/bioethicists. When presented with the list of actual or potential risks, it is possible that PLWHIV display more aversion to clinical research risks [3]. Third, each key informant was only interviewed once and longitudinal data collection would have allowed us to derive evolving perceptions of risks and benefits. Fourth, the interviews focused on HIV cure research in general, and more research will be needed regarding perceived risks and benefits of specific modalities or studies. Fifth, since the sample represented a very small subset of people interested in HIV cure research, the data are not representative of the population of PLWHIV, clinician-researchers, and policy-makers/bioethicists in the United States. Finally, our findings are not applicable to contexts outside of the United States, particularly resource-limited settings, where health systems, access to treatment and care, capacity for HIV cure clinical research and values may differ. Despite these limitations to generalizability, the findings reported therein have internal validity with respect to the categories of key informants who participated in the study. The exploratory results may provide a stepping stone for future research using more robust methodologies to characterize risk perceptions across the translational research continuum and their impact on decision-making and determinations to move HIV cure investigational interventions forward [32].

## Conclusion

In sum, while perceived risks/benefits may differ from actual risks/benefits, it is important to understand the perspectives of various stakeholders involved in HIV cure research, since these have consequences for the effective and ethical implementation of HIV cure research, including how study protocols and informed consent forms are designed. The possibility of exposing PLWHIV to anything that harbors unacceptable risk appears unethical, and HIV cure research protocols should have clear safety and tolerability criteria that can be acted upon promptly during the conduct of HIV cure research. Further, because HIV cure research remains a relatively recent endeavor, we have a unique opportunity to develop high ethical standards surrounding risks and benefits. Policy-makers/bioethicists have a role to play in creating policies that address the category of unacceptable risks in HIV cure research. It is also crucial to manage expectations around what the science can deliver in early-phase experiments [1], and to provide adequate education to potential study participants. While our study attempted to bridge social sciences with the biomedical research on HIV cure [42][43], more formative research will be needed to understand the evolving public discourse on specific types of HIV cure studies, and this will need to involve the close collaboration of biomedical researchers,social scientists and community representatives. Emerging results around perceived risks and benefits of HIV cure research can serve as an empirical foundation for knowledge translation and community engagement strategies to support the long-term development of a cure with an acceptable risk and benefit profile. Finally, community and participant confidence regarding the safety and acceptability of a cure should be a compelling driver for discovery and progress towards such a cure.
